# “I am Here”—The Importance of Caring Touch in Intensive Care. A Qualitative Observation and Interview Study

**DOI:** 10.1177/08980101231198723

**Published:** 2023-09-21

**Authors:** Jessica Tengblad, Fanny Airosa, Liza Karlsson, Johanna Rosenqvist, Carina Elmqvist, Ann-Christin Karlsson, Maria Henricson

**Affiliations:** Ryhov County Hospital; Karolinska Institute; Växjö County Hospital; Department of Research and Development, Region Kronoberg Department of Health and Caring Sciences, Linnaeus University; Uppsala University/Campus Gotland; 1802 Faculty of Caring Science, Work Life and Social Welfare, University of Borås Jönköping Academy for Improvement of Health and Welfare, Jönköping University

**Keywords:** adults, group/population, families, touch therapy, intensive care, qualitative content analysis, healthcare professionals

## Abstract

**Purpose:** The purpose of the study was to illuminate the experience of caring touch in intensive care from the perspectives of patients, next-of-kin, and healthcare professionals. **Design and Method:** This study was explorative, and data were collected through qualitative observations (*n* = 9) with subsequent interviews (*n* = 27) at two general intensive care units. An inductive approach was embraced to be open-minded to the participants’ experiences. **Findings:** The results are presented in one generic category—caring touch creates presence—which generated five subcategories: to touch and be touched with respect, touch as guidance and communication, touch causes suffering, touch creates compassion, and touch creates security. **Conclusion:** When the ability to communicate with words is lost, it is body language that reveals what a person is trying to express. Nurses create a way of being present with the patients by touching them, to communicate I am here for you. Caring touch is a tool to show compassion and respect and to protect the integrity of the lived body. The caring touch is soothing and comforting for the patient and next-of-kin and creates security. It also helps to awaken the motivation to get healthy, which is needed in an environment that is foreign.

## Background

Holistic nursing refers to nursing practice that has healing the whole person as its goal (Cavan Frisch & Rabinowitsch, 2019; Frisch et al., 2000). In the philosophy and practice of holistic nursing, one core value includes self-care, self-reflection, and self-development. It is also crucial to nurses’ ability to see patients as a whole person and provide holistic, compassionate care (AHNA, 2022; Blaszko Helming et al., 2020). Holism requires a progressive nurse–patient relationship based on openness, equality, and mutuality, where the patient's family is included (Cavan Frisch & Rabinowitsch, 2019). Might a holistic approach in a high-tech intensive care unit (ICU) provide an excellent environment for patients healing?

### To be Cared for in Intensive Care

Intensive care is a foreign environment for patients that is unknown, incomprehensible, and sometimes scary and frightening. Advanced technology is a necessary part of such care (Foster & Hawkins, 2005; Rose et al., 2019; Wilkin & Slevin, 2004), but can affect the circadian rhythm and cause immobilization, pain during treatment and, for intubated patients, an inability to communicate, which can in turn contribute to stress and suffering. Additional stressors include invasive procedures, loss of privacy, separation from family, sleep disturbances, and constant light and sound disturbances (Reich et al., 2010). When healthcare is technologically advanced, the need for holistic nursing with human contact and closeness must be considered (Tingsvik et al., 2018). It is a challenge for healthcare professionals (HCPs) to strike a balance between holistic nursing and technology at ICUs, as patients are often connected to medical equipment that is important for highly specialized care but makes them unable to communicate their needs (Price, 2013; Tunlind et al., 2015).

### The Presence of HCPs and Next-of-Kin

In Scandinavian ICUs, patients have an assistant nurse or registered nurse bedside 24/7, which can relieve their anxiety, fear, and loneliness. The presence of an HCP promotes a patient's sense of stability, balance, and safety (Baumgarten & Poulsen, 2015). In addition, next-of-kin is described as playing an important role for patients, as the family becomes involved in care when a person is critically ill (Bailey et al., 2010; Blom et al., 2013; Valle & Lohne, 2021). Next-of-kin involvement is helpful, as many patients cannot communicate verbally. For next-of-kin, not knowing what is going to happen can be frightening, so it is of great importance to involve them in the holistic care process (Blom et al., 2013; Elmqvist & Frank, 2015). This can also provide hope and keep both patients and families going at a difficult time. When next-of-kin sees that a patient responds positively to treatment provided by HCP, this raises their hopes and relieves their suffering (Valle & Lohne, 2021). The opposite of hope is disconnectedness and a sense of emotional vulnerability. Next-of-kin feels disconnected when HCP disengage from them emotionally and physically (Wong et al., 2018).

### Caring Touch

Caring touch is a part of holistic nursing at an ICU, where the goal is to heal the whole person; closeness and touch are basic human needs. Caring touch takes place in interactions between individuals and can be of crucial importance in ICU HCPs’ communication with patients and their next-of-kin (Pedrazza et al., 2018; Picco et al., 2010; Wilkin & Slevin, 2004). A central aspect of intensive care is that the patient is seen as an equal partner in care and that the partners show mutual respect for each other's knowledge. The patient should be seen as a person with resources and abilities, not just a passive recipient of care (Tingsvik et al., 2018). Person-centered and life-oriented ethics mean that the professional and the patient collaborate (Goldfarb et al., 2017).

In healthcare, caring touch can have three different purposes, which are described by Pedrazza et al. (2018) as task-oriented, emotional, and/or intended to create physical comfort. *Task-oriented* touch might involve turning the patient and changing the linens, and *emotional* touch aims to provide support to the patient, whereas *physical comfort* can, for example, be a gentle massage. Task-oriented and emotional touch have been observed in the present study. Physical contact is necessary for helping patients in daily care (Picco et al., 2010; Wilkin & Slevin, 2004). Caring touch can be expressed in many ways, and can, for example, give a patient a sense of presence in a meaningful context and help them to feel seen, accepted, and confirmed (Ozolins et al., 2015). When HCPs perform clinical tasks, caring touch can be interpreted as an expression of compassion, warmth, care, empathy, and presence (Kelly et al., 2018), which means that the patient becomes more aware of himself/herself through touch (Ozolins et al., 2015). The patient may also interpret touch in a different way than intended, which may overshadow the potential benefits. Therefore, HCPs need to be aware of each patient's personal boundaries and actively decide if, when, and how to touch them (Karlsson et al., 2022; Kelly et al., 2018; Ozolins et al., 2015). Caring touch is not synonymous with healing touch or therapeutic touch, which includes a set of standardized techniques that clear, energize, and balance the human energy system (Bulette Coakley & Duffy, 2010; Davis et al., 2020).

### Lifeworld Approach

The theoretical framework for the present study involved a caring perspective based on a lifeworld approach. A holistic care with a lifeworld approach means paying attention to how health, illness, suffering, and well-being are experienced by the individual. Because the patient is perceived as the foremost expert on their situation, a holistic view, and the patients' and next-of-kin's participation, is realized with a lifeworld approach.

According to Dahlberg et al. (2008), we can never escape the lifeworld, the complex, qualitative, and lived reality that is there for us whatever we do. We understand the world through our bodies, through its possibilities and its limitations. The lived body is a unity of body and soul and can further be described as a carrier of the person's life-history and essential meaning—the home of its existence. To the individual, the body is obvious. When a patient's lived body changes due to severe illness, and he or she needs intensive care, it brings about a change in both the patient's and the next-of-kin's lifeworld (Dahlberg et al., 2008; Merleau-Ponty, 2018). Seen from a caring perspective, the human body is a nest of health and suffering, and this perspective makes us aware that caring is a meeting of two opposites. The HCPs must have the ability to see that particular individual experience of being ill and suffering. When patient is vulnerable in their suffering, they need to meet fellow human beings who show openness and readiness to accept the sufferer's suffering, which is characteristic of a caring relationship (Lindström et al., 2014; Morse, 2001). The patient can sense the HCP's intention and attitude to care by reading body posture, gaze, addresses, touch, and the caring actions performed.

Previous research regarding caring touch in intensive care is scant; only a few studies have been found. The studies performed have focused on touch as a treatment, such as massage or tactile touch (Henricson et al., 2009; Jagan et al., 2019). By examining how patients, next-of-kin, and HCPs experience caring touch in intensive care, it is possible to improve holistic nursing and contribute to an increased understanding and awareness of its caring effects.

## Aim

The aim was to illuminate the experience of caring touch in intensive care from the perspectives of patients, next-of-kin, and HCPs.

## Method

### Study Design

This study was explorative, and data were collected through qualitative observations and subsequent interviews with patients, next-of-kin, and HCPs. An inductive approach was embraced to be open-minded to the participants’ experiences (Polit & Beck, 2020).

### Setting and Participants

The study was conducted at two general ICUs at county hospitals in Sweden that had 6–8 beds and a 1:1.4 nurse–patient ratio. The wards consisted of single-, double-, and four-bed rooms. Next-of-kin and visitors were permitted to access 24 hr. Variation in the gender and age of participants was sought, as well as variation in patients cared for in single rooms and multibedrooms (Polit & Beck, 2020). Inclusion criteria were participants >18 years cared for in an ICU at least 24 hr before inclusion. Exclusion criteria were patients under the influence of sedatives, diagnosed with psychosis, suffering from trauma or head injury, or who had cognitively impaired next-of-kin (Tables 1 and 2). Next-of-kin referred to the person whom the patient defined as a relative and HCPs the nurses who were in the room during the current situation. In total, nine patients, four next-of-kin, eight nurses, and eight assistant nurses, in a total of 27 interviews, were included in the study.

**Table 1.** Summary of Participants in Observations and Interviews

	Informant	Gender	Age	Numbers of years in the profession	Number of years in intensive care unit (ICU)	Conducted interview
Observation 1	Patient	Male	74			No
	Next-of-kin	Female	70			Yes
	Intensive care nurse	Female	46	25	4 months	Yes
	Assistant nurse	Female	44	26	3	Yes
Observation 2	Patient	Female	68			Yes
	Intensive care nurse	Female	56	38	30	Yes
	Assistant nurse	Female	55	3,5	10 months	Yes
Observation 3	Patient	Male	64			No
	Next-of-kin	Female	38			Yes
	Assistant nurse	Female	46	12	3	Yes
Observation 4	Patient	Male	74			Yes
	Next-of-kin	Female	69			Yes
	General nurse	Male	28	4	1, 5	Yes
	Assistant nurse	Female	28	9	7 months	Yes
Observation 5	Patient	Female	83			Yes
	Next-of-kin	Female	59			Yes
	Intensive care nurse	Female	30	8	1 ½ months	Yes
Observation 6	Patient	Male	54			Yes
	Intensive care nurse	Female	25	2	3 months	Yes
	Assistant nurse	Female	42	24	14	Yes
Observation 7	Patient	Female	70			Yes
	Intensive care nurse	Female	57	35	30	Yes
	Assistant nurse	Female	63	25	14	Yes
Observation 8	Patient	Male	79			Yes
	Intensive care nurse	Female	54	32	32	Yes
	Assistant nurse	Female	22	4	1	Yes
Observation 9	Patient	Male	78			Yes
	Intensive care nurse	Female	41	15	10	Yes
	Assistant nurse	Female	36	10	6	Yes

**Table 2.** Parameters in the Data Collection

Parameter	Average
Age of informants	53
Patients hospitalized number of days	3, 5
HCP number of working years	17
HCP number of years in ICU	9
Interview, length in minutes	18
Observation, length in minutes	27

*Note*. HCP = healthcare professional; ICU = intensive care unit.

### Data Collection

The study was conducted as a qualitative observational study with subsequent semistructured interviews (Polit & Beck, 2020) to provide a deeper understanding of the phenomena. The observer was present and made continuous observations of what was happening, with a chance to see the same things as those observed. The opportunity to interview the patients, next-of-kin, and nurses after observation increased the possibility of ensuring that the observer had interpreted the observed situations correctly (Polit & Beck, 2020).

An observation protocol, based on the caring concepts of suffering, well-being, caring relationship, and lived body (Dahlberg et al., 2008; Merleau-Ponty, 2018), was used to ensure that the observation was carried out in the same way and that information related to the purpose was collected. The observations took place during the daytime at the patient's bedside: four in one four-bedroom and five others in single rooms. The first author conducted all observations and interviews at Hospital A, and two master's students did the same at Hospital B. Situations, such as nurses helping a patient with personal hygiene and mobilization both bedside and in bed, the evaluation of pain relief, breathing exercises or testing a neck collar, were observed for approximately 30 min. The informants were aware that the study was being carried out and that the observer would not actively participate in any care situations. The observations were followed by individual semistructured interviews, which were introduced with the same question: *How did you experience the caring touch?* The interviews then continued with questions based on the field notes in the observation protocols, in as close connection as possible to the observation—depending on the status of the patient, next-of-kin, and HCPs. The patients were interviewed at their bedside, with folding walls used to screen off the space, while next-of-kin and HCPs were interviewed in a private conference room. The interviews were recorded and lasted between 15 and 25 min. After observations, two patients opted out of participating in interviews.

### Data Analysis

The data analysis was carried out in accordance with Elo and Kyngäs’s (2008) qualitative inductive content analysis method. The interview transcripts (48 pages for Hospital B, 64 pages for Hospital A), observation protocols (18 pages), and field notes (5 pages) constituted the units of analysis. Those parts of the observation protocols that were quantifiable were quantified (Table 3). As a first step, the observation protocols, field notes, and transcribed interviews were read by the first author several times to grasp the text as a whole and gain a deeper understanding thereof. In the next step, the units of analysis were coded based on the aim of the study. The first and last author compared and discussed the codes in order to reach a consensus before the codes were grouped into preliminary categories. By moving back and forth between the units of analysis, codes, and preliminary categories, the authors identified the subcategories and the links between them based on similarities and differences. To increase trustworthiness, the remaining authors read the preliminary categories and subcategories and compared and adjusted the codes from the transcript, before the first author abstracted the subcategories into one generic category.

**Table 3.** The Results According to the Observation Protocol

Observations parameters	(*n* = 9)
Information before touching Hospital A and B	8
Reduced suffering Hospital A	3
Increased well-being Hospital A	4
Improved caring relationship Hospital A	3
Respect for the patient's integrity and lived body Hospitals A and B	7

### Ethical Considerations

The study was conducted following The International Council of Nurses’ Code of Ethics (2021). All participants were informed verbally, and received written information about the nature and purpose of the study and that the data would be treated in strict confidence. They were also informed about their right to withdraw from the study at any time without explanation or consequences. They were also informed that they would remain anonymous in the presentation of the results. Written informed consent was obtained.

## Results

The results are presented in one generic category—caring touch creates presence—which generated five subcategories: to touch and be touched with respect, touch as guidance and communication, touch causes suffering, touch creates compassion, and touch creates security. Both registered nurses and assistant nurses are referred to as “nurses” and “healthcare professionals” in the results. When quotes are used, P stands for patient, NoK stands for next-of-kin, and N stands for nurses.

### Caring Touch Creates Presence

Caring touch gives the patient a feeling of being respected as a person, with their body and personal integrity considered. In critically ill patients who have difficulty communicating, communication and guidance took place through touch. HCPs and next-of-kin touched a patient to show that they were present and conveyed security and compassion.

#### To Touch and be Touched With Respect

Touching and being touched with respect was important. Respect for the body, personal integrity, and independence, when a patient is critically ill and dependent on healthcare, were perceived as important by HCPs, patients, and next-of-kin. Touch was described by next-of-kin, patients, and HCPs as careful, soft, gentle, and respectful. The caring touch could be perceived as harsher if HCPs were stressed and more pleasant if they had more time for nursing. During the observations, it was noted that the patients’ integrity was protected and treated with respect. Patients’ bodies were touched with respect and HCPs did not expose them during nursing. None of the patients reacted negatively to touch during care provision.

In the observed situations, some unnecessary use of gloves was noted. One example was that HCPs used gloves when touching a patient's arm. The purpose of this touch was to support the patient during mobilization. One patient said:

P. “Skin on skin is softer, so the gloves can make it so it's a bit stiff at times, and yeah, but, it went well and yeah … // … when you have bare hands, it's much softer … // … they have gloves and that kind of thing nowadays …”

Experienced HCPs said that they could see when a patient did not appreciate touch by looking at the patient's facial expressions and body language, which showed if touch was unpleasant. Out of respect for each patient's body and integrity, HCPs avoided unnecessary touching.

#### Touch as Guidance and Communication

Touch was used as guidance and communication by HCPs and next-of-kin, who would often touch patients with the intention to communicate. Touching would take place on the patient's arm or hand or by gently stroking the face, to tell the patient: “I am here.” It was also a way to get a patient's attention when HCPs wanted to communicate something.

The nurses used touch as communication in connection with mobilization and nursing measures, so that the patient would understand where the HCPs were.

NoK. “Yeah, they went ahead and showed him how to get up on the side, so that he could sit. Yeah. I thought that they, they were really good at it. Gentle and, like, and waited until he had maybe gotten it right, that he was sitting up. Yeah. That's what I thought, anyway.”

During the observations, the nurses touched the patients to provide guidance on how they could be involved in their own care. Touch could give patients clarifying instructions on how a mobilization would proceed. Nursing staff might touch a patient's shoulder or arm to show which direction the patient should turn during mobilization. The patients felt that the touch provided safety and security when moving in and out of bed or during toilet visits. Touch thus became a supportive aid.

P. “I don’t really mind … It's something that I can accept entirely … they have to … umm … touch me, move me, or do things that have to be done in order to examine me or treat me … Their touching me is not something I see as a problem, it's completely normal.”

Touch was considered something the patient could expect in nursing. It also emerged that previous care experiences played a role in how patients reflected on touch. A familiarity with being touched could develop, which led to less reflection on how touch was being given.

#### Touch Causes Suffering

The HCPs also said that touch could lead to increased suffering for patients. For example, it was felt that a patient's integrity could be violated, which might lead to the patient not accepting the performed touch. When this happened, the HCPs felt that patients suffered as a result of touching. Such touching was described as painful, with examples being when an injured area was being examined or when the patient was being turned over. It was important to evaluate each situation and interpret how receptive patients were to being touched in order to avoid misunderstandings and discomfort. This led, among other things, to HCPs being more restrictive with caring contact with anxious patients.

It was also described that refraining from touch could give rise to suffering among HCPs. This suffering was of an existential nature and arose when HCPs did not dare touch patients who were in a difficult situation or experienced great suffering. The patients experienced that touch could create suffering when an HCP's hands were too cold or when the touch occurred in an area where they had pain. The patients also described that touch could be perceived as violating privacy. This was mainly when it came to touching intimate areas of the body and when they felt that they did not receive any information about what was going to happen.

P. “Yeah, it was okay … or … or, yeah, no … it wasn’t okay. I thought it was … They could have said that like … ‘Now, we’ll be washing you here and now we’ll be washing you there’ … but they didn’t, they were silent, both of them … That was a bit difficult … Because it's a pretty intimate thing.”

#### Touch Creates Security

Touch creates security, conveys compassion, relieves suffering in patients at the ICU, and could be used to massage or steady a patient's body to divert pain and improve well-being. HCPs felt that security could be created through touch and being near patients in the ICU.

N. “a little because you probably feel, or, yeah, that they feel vulnerable, our patients, lying in bed, and you just, you can’t just turn them over and do what you’re supposed to, but you hold on a little, yeah, you hold tight, you might say, and not in that sense, but just so that they feel a bit safer … // Yeah, I imagine that that makes you feel a bit safer. In what we are doing …”

Caring touch was performed by HCPs, for example, placing a hand on a patient's arm or shoulder and thus supporting the patient. Patients stated that they felt safe thanks to caring touch. Touch could also relieve the suffering of patients when they were in an unpleasant or stressful situation. The HCPs felt that touch on these occasions could mark their physical presence and communicate calm and security. This type of touch was often performed while a verbal communication was taking place with the patient. The HCPs experienced that the calm and security was reflected in patients, who became more relaxed and showed less physical anxiety.

N. “Like for instance if you are extubating, you hold their hand and try to calm them down and talk and try to hold their hand and be present, since you know that when you extubate and stop sedation, the first thing that comes back is hearing and you can’t move that much, but if they feel that you are there, it makes things a bit calmer.”

Patients felt that when they received adequate information about what was going to be done and where touch was going to take place, it could lead to security and make the situation easier to handle, which could in turn reduce anxiety and suffering. The information they received in connection with touch make the patients see the nurses as more professional, which strengthened their sense of security.

#### Touch Creates Compassion

Touch was considered soothing and comforting. Patients described that they felt the compassion of the HCPs through their touch. For HCPs, touch was a way to show empathy, that they wanted to help the patients emotionally and support them in a vulnerable situation.

P. “… and then touch can be great, if you get sad or something, that the nurses touch your shoulders or something for me, and that you feel you have permission to cry a bit. After a while, when it's passed, you can get yourself together and so on, at least most of the time, so, yeah …”

Patients described that touch was there to comfort and help them and that it could give them emotional support during the care period. Caring touch helped a patient relax in connection with the nursing and procedures being done in the ICU and provided a source of calm and comfort. Next-of-kin said that they often held their relative's hand or hugged them, which was experienced as calming and comforting.

Close physical contact from HCPs and/or next-of-kin, in the form of a firm handhold, made patients feel comfortable and calm when in a vulnerable situation. Touch was used by HCPs to create contact, but also to improve the care relationship with a patient.

## Discussion

In this study, both HCPs and patients emphasized the importance of all types of touch being preceded by information to patients, which creates a sense of safety and security. According to all the participants, care should be provided with respect, which is one of the cornerstones of Swedish healthcare. From the patient's perspective, it was considered important to preserve integrity and show respect for the whole person. Previous research in intensive care has shown that there is a risk that HCPs violate patient integrity if patients are not given adequate information ahead of a caring contact (Moen & Nåden, 2015; Nyholm & Koskinen, 2017). The next-of-kin's experiences were of seeing their relatives treated with respect, with HCPs able to provide care in a respectful way. Respect for patient integrity is a recurring theme in Ozolins et al.'s (2015) research about caring touch. According to Moen and Nåden (2015) and Nyholm and Koskinen (2017), it is important that touching in nursing situations is performed in such a way that it does not affect patient integrity, since patients are already in a vulnerable situation. Our lived body is the basis for our understanding of the world, and Merleau-Ponty (2018) believes that the person should be considered as a whole. Through touch, a person can be perceived as an integral unit, where body and soul are combined (Ozolins et al., 2015). Touch must relate to the lifeworld of the person receiving that touch and what that person prefers when it comes to caring touch.

The results showed that next-of-kin and HCPs used touch as guidance and to reinforce verbal communication. When the ability to communicate with words was lost, body language revealed what a person was thinking or trying to express. Tingsvik et al. (2018) have emphasized that patients who are cared for in a mechanical ventilator cannot communicate verbally, which increases demands on HCPs to find alternative ways of communicating. It is important that HCPs are aware of a patient's efforts to communicate nonverbally and that they interpret facial expressions and body language (Karlsson et al., 2012).

In the present study, touch was found to create security and compassion for patients and next-of-kin. Touch was a way for HCPs to show compassion, be calm, and present, and give patients security. Next-of-kin often held their relative's hand or hugged them, which was experienced as comforting. Casarini et al. (2009) have confirmed that patients and next-of-kin have a need for security and closeness in an environment that is foreign and filled with technology. HCPs can make next-of-kin feel safe through touch as a way of showing presence and that there is someone there to share their experiences with (Karlsson et al., 2022).

Tingsvik et al. (2018) indicated that when patients are anxious, they appreciate the presence of their loved ones and experience touch and scents that are familiar to them. If a touch is soft and empathetic, it can convey calm and comfort to the patient, which is also confirmed in Ozolins et al.’s (2015) research. Furthermore, Kelly et al. (2018) have suggested that touch can express warmth, compassion, and peace to patients. Through touch, emotions can be conveyed in a way that overcomes the absence of words (Determeyer & Kutac, 2018).

There can be individual differences in how comfortable HCPs are with touch. Airosa et al. (2016) and Andersson et al. (2007) highlighted the importance of a supportive environment and an individual internal balance for HCPs to feel comfortable with touch. There is a potential to improve the quality of nursing by raising awareness of the importance of touch among HCPs, which might reduce the unnecessary use of gloves. The results of the present study showed that patients experienced a difference in the quality of the touch depending on whether gloves were used. Routasalo (1996) confirmed that patients preferred bare-hand contact over gloved-hand contact, which created a feeling of coldness. Furthermore, Karlsson et al. (2022) underline that touching a patient without gloves is important to make better contact.

### Methodological Considerations

To improve trustworthiness, the components *credibility, objectivity, dependability,* and *transferability* from Elo et al.’s (2014) checklist were used. *Credibility* was considered strong since the nurses at hospitals A and B recruited patients without oversight by the first author. To establish *objectivity,* the first author and the master's students, who conducted the observations and interviews and performed the analysis, dealt with their preunderstandings through peer debriefing and communication with the other authors on the research team. The experiences of the topic were from the first author's professional role as an intensive care nurse and from the students in the master's program in intensive care. *Objectivity* was achieved by using the same protocol in all observations, recording the interviews, transcribing them verbatim, and presenting quotations from the participants. Furthermore, the fact that the same author and the master's students conducted the observations and the subsequent interviews also strengthened objectivity. To enhance the *dependability* of the content analysis, the participants were chosen to represent a range of ages, genders, and professional experiences. A limitation was that the presence of a researcher as an observer over several days might have affected the results, since the participants became aware of what was being observed. The setting, selection of participants, data collection, and analysis process were described in detail to facilitate comparison with other studies, which strengthened *transferability* (Elo et al., 2014).

## Implications for Research and Practice

In an ICU where patients are treated in a highly technologically advanced environment, caring touch always needs to be a part of holistic nursing. The result of the study shows that caring touch is used as well as a tool to help the patient with guidance and communication, such as creating security and showing empathy. The HCP's knowledge and education in caring touch have an impact on the patient's and the next-of-kin's care experiences. The challenge in clinical practice is to create a tolerant and conscious environment that promotes touch, which for the patients entails holistic care. The understanding and awareness of the importance of caring touch for patients, their next-of-kin, and HCPs need to be included in nursing education. Furthermore, we propose continuous reflections in the college regarding the ICU to raise awareness of the importance of caring touch and exchange experiences.

## Conclusion

When the ability to communicate with words is lost, it is body language that reveals what a person is trying to express. Nurses create a way of being present with the patients by touching them, to communicate *I am here for you*. Caring touch is a tool to show compassion and respect and to protect the integrity of the lived body. The caring touch is soothing and comforting for the patient and next-of-kin and creates security. It also helps to awaken the motivation to get healthy, which is needed in an environment that is foreign and full of advanced and frightening technology.
